# Coexistence of Ferroelectricity and Ferromagnetism in Fullerene‐Based One‐Dimensional Chains

**DOI:** 10.1002/advs.202301265

**Published:** 2023-05-10

**Authors:** Yang Zhao, Yu Guo, Yan Qi, Xue Jiang, Yan Su, Jijun Zhao

**Affiliations:** ^1^ State Key Laboratory of Structural Analysis Optimization and CAE Software for Industrial Equipment Dalian University of Technology Dalian 116024 P. R. China; ^2^ School of Physics and Materials Engineering Dalian Minzu University Dalian 116600 P. R. China

**Keywords:** ferroelectricity, ferromagnetism, fullerene, one‐dimensional, semiconductor

## Abstract

One‐dimensional (1D) magnetoelectric multiferroics are promising multifunctional materials for miniaturized sensors, actuators, and memories. However, 1D materials with both ferroelectricity and ferromagnetism are quite rare. Herein, using first‐principles calculations, a series of fullerene‐based 1D chains, namely U_2_C@C_80_‐M (M = Cr, Mn, Mo, and Ru) 1D chains with both ferroelectric (FE) and ferromagnetic (FM) properties is designed. Compared to individual U_2_C@*I*_h_(7)‐C_80_, the spontaneous polarization (Ps) in 1D chains is enhanced by about two to four times owing to the interaction between U_2_C@*I*_h_(7)‐C_80_ fullerene and M (M = Cr, Mn, Mo, and Ru) atoms. Meanwhile, the introduction of transition metal atoms dopes electrons into U's 5*f* orbitals, leading to numerous intriguing magnetic properties, such as U_2_C@C_80_‐Cr and U_2_C@C_80_‐Mo as 1D ferromagnetic semiconductors, U_2_C@C_80_‐Ru as 1D ferrimagnetic (FiM) semiconductor, and U_2_C@C_80_‐Mn as 1D antiferromagnetic (AFM) semiconductor. Excitingly, it is found that magnetic ordering and electrical polarization can be modulated independently by linking different transition metal atoms. These findings not only broaden the range of 1D multiferroic materials, but also provide promising candidates for novel electronic and spintronic applications.

## Graphical abstract

This work designs a series of fullerene‐based 1D chains with excellent ferroelectric and ferromagnetic properties. Due to the cooperative effect between fullerenes and metal linkers, their spontaneous polarization is higher than freestanding U_2_C@*I*_h_(7)‐C_80_ by about two to four times. Meanwhile, robust 1D ferromagnetic/antiferromagnetic semiconductors with high Curie or Neel temperature are obtained.

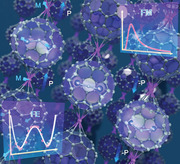

## 1 Introduction

Multiferroicity mainly refers to a class of materials with ferromagnetic/antiferromagnetic (FM/AFM), ferroelectricFE, and ferroelastic (FA) properties, and their order parameters (spin polarization, electric polarization, and strain) can be switched by external stimuli such as magnetic fields, electric fields and mechanical loading. Since the characteristics of dual stability and reversible phase transition resemble the basic logic units “0” or “1” in computer, multiferroic materials can be potentially used to fabricate logic switching and non‐volatile memory storage devices.^[^ ^1^, ^2^, ^3^ ^]^

Motivated by the urgent need for miniaturization of modern electronic devices, one‐dimensional (1D) or even single‐molecule scale materials with excellent ferroelectric, ferromagnetic, or ferroelastic properties have become the focus of intensive research. Among them, endohedral metallofullerenes hold great promise in magnetic storage and electrical devices due to relatively mature synthetic techniques and unique cage structure.^[^ ^4^ ^]^ For example, encapsulating Gd atom in the C_82_ cage could realize a low‐temperature single‐molecule electret that shows gate‐controlled switching between two electronic states.^[^ ^5^ ^]^ Using the scanning tunneling microscope‐break junction technique, room‐temperature logic‐in‐memory operations of two‐terminal single endohedral metallofullerene Sc_2_C_2_@*C*_s_(hept)‐C_88_ devices were fabricated, in which reversible encoding and storage of digital information had been realized by quasi‐permanent dipole [Sc_2_C_2_] group in the fullerene cage.^[^ ^6^ ^]^ In addition, by inserting DySc_2_N@C_80_ into single‐wall carbon nanotubes, Shinohara et al. achieved the enhancement of single‐molecule magnet properties of DySc_2_N@C_80_ due to the suppression of quantum tunneling of magnetization, also indicating that embedded fullerenes are favorable candidates to design memory storage information units in the future.^[^ ^7^ ^]^

However, there remain grand challenges in the exploration of ferroelectric or ferromagnetic materials based on endohedral metallofullerene, such as high stability, intrinsic spontaneous electrical polarization with suitable transition energy barrier and magnetic bistability. Experimentally, actinide endohedral fullerenes have been successfully fabricated,^[^ ^8^, ^9^, ^10^, ^11^, ^12^ ^]^ in which the 5*f* orbitals of actinide elements feature strong localization and spin–orbit interaction, and the unique cavity structure of fullerene could provide an independent inert space for highly active actinide compounds. Thus, the unique multiple bonds, including actinide metal‐metal/metal‐nonmetal bonds in cages and inter‐orbital hybridization, are expected to induce excellent ferroelectric, piezoelectric, and magnetic properties and solve the current bottleneck problem in the miniaturization of electronic devices. However, most actinide single‐molecule magnets are often accompanied by significant quantum tunneling relaxation phenomena, which reduce the effective energy barrier of the magnet and the relaxation time.^[^ ^13^ ^]^

In this paper, we elaborately selected 3*d*/4*d* transition metal atoms to bridge experimentally synthesized diuranium carbide endohedral metallofullerene U_2_C@*I*_h_(7)‐C_80_,^[^ ^12^ ^]^ to construct a series of stable 1D chains, namely U_2_C@C_80_‐M (M = Cr, Mn, Mo, and Ru), in which the 3*d*/4*d* transition metal atoms are beneficial to optimize the coordination environment of actinide ions and promote the magnetic properties.^[^ ^14^, ^15^, ^16^, ^17^, ^18^ ^]^ Our ab initio calculations demonstrate that these fullerene‐based 1D chains exhibit excellent intrinsic ferroelectricity, with spontaneous electric polarization and low ferroelectric switching barrier due to breakdown of the centrosymmetry of U_2_C unit in C_80_ cage. Meanwhile, they show distinct magnetic properties and behave as ferromagnetic semiconductors, antiferromagnetic semiconductors, or ferrimagnetic semiconductors, depending on the transition metal element. Therefore, our findings achieve independent regulation of magnetic order and electrode by connecting different transition metals, thereby expanding the application of endofullerene in the field of multiferroics.

## 2 Results and Discussion

U_2_C@*I*_h_(7)‐C_80_, as a building unit with ferroelectric and ferromagnetic properties, was selected for cluster assembly mainly due to two points. First, uranium atoms, thanks to the strong spin‐orbit coupling and multiconfigurational nature of the ground state that generates strong magnetic anisotropy, are powerful candidates for obtaining excellent magnetic materials. Second, the encapsulated [U_2_C] trimer has highly polarized U–C interaction with a flexible U–C–U angle ranging from 120° to 180°, which can realize polarized and unpolarized states through molecular vibrations.^[^ ^12^ ^]^ Moreover, 3*d*/4*d* transition metal atoms as linkers^[^ ^17^, ^19^, ^20^, ^21^ ^]^ could further modulate the ferroelectric and ferromagnetic properties of endohedral metallofullerene U_2_C@*I*_h_(7)‐C_80_ via *π*‐*d*‐*π* hyperconjugation.^[^ ^22^, ^23^, ^24^, ^25^, ^26^, ^27^, ^28^ ^]^

**Figure 1:**
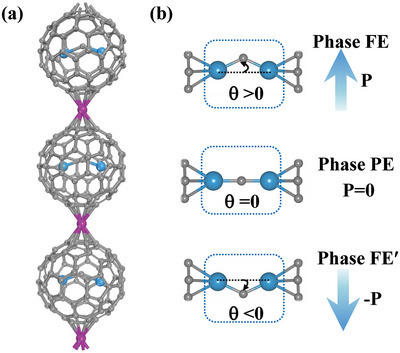
a) U_2_C@C_80_‐M (M = Cr, Mn, Mo, and Ru) 1D chains, where gray, blue, and purple denote C, U, and M atoms. b) Schematic side views of the two distorted degenerate polar structures (FE and FE′) and the high‐symmetry nonpolar phase (PE).

As shown in **Figure** **1**a, the designed U_2_C@C_80_‐M 1D chains are composed of transition metal M atoms (M = Cr, Mn, Mo, and Ru) connected with U_2_C@C_80_ fullerenes. We first evaluate their thermodynamic stability by calculating the formation energy (*E*_f_) as follows

(1) $$E_{f}=E_{U_{2}C@C_{80}-M}-E_{U_{2}C@I_{h}-C_{80}}-E_{M}$$

where $E_{U_{2}C@C_{80}-M}$, $E_{U_{2}C@I_{h}-C_{80}}$, and *E*_M_ are the energies of U_2_C@C_80_‐M 1D chain, U_2_C@*I*_h_(7)‐C_80_ metallofullerene and transition metal M atom, respectively. As summarized in **Table** **1**, the calculated *E*_f_ are −3.21, −3.26, −3.67, −3.40, and −4.49 eV for M = Cr, Mn, Mo, and Ru, respectively, indicating experimental synthesis feasibility of these 1D chains. Meanwhile, taking the U_2_C@C_80_‐Mo as a representative (Figure S1, Supporting Information), ab initio molecular dynamics (AIMD) simulation shows that the system can maintain its 1D chain structure at temperature up to 600 K, indicating good thermal stability.

**Table 1:** Calculated lattice parameters *a*, U−C−U angle *θ*, formation energy *E*_f_, and band gap $E_{g}^{\mathrm{HSE}}$ of U_2_C@C_80_‐M 1D chains and individual U_2_C@*I*_h_(7)‐C_80_ metallofullerene

	*a* [Å]	*θ* [°]	*E*_f_ [eV]	$E_{g}^{\mathrm{HSE}}$ [eV]
U_2_C@*I*_h_(7)‐C_80_	–	145.6	–	0.55
U_2_C@C_80_‐Cr	11.92	133.3	−3.21	0.90
U_2_C@C_80_‐Mn	11.75	129.0	−3.26	1.43
U_2_C@C_80_‐Mo	11.44	141.5	−3.40	1.07
U_2_C@C_80_‐Ru	11.44	145.1	−4.49	1.15

It is noteworthy that the optimized geometry of individual U_2_C@*I*_h_‐C_80_ is close to the experimental single‐crystal data (U—C bond length: 2.04 Å vs 2.03 Å; U–C–U angle: 145.6° vs 142.8°; U…U distance: 3.90 Å vs 3.85 Å).^[^ ^12^ ^]^ After forming 1D chain, the enclosed U_2_C units still retains the notably buckled structure, in which the C atoms break U–C–U line plane of the centrosymmetric phase *Pmmm* to reach polar phase *Pmm*2 in Figure 1a. The electronic band structure of U_2_C@C_80_‐M chains are comprehensively investigated by Heyd–Scuseria–Ernzerhof hybrid function (HSE06) calculations in Figure S2 (Supporting Information). Compared with the highest occupied molecular orbital‐lowest unoccupied molecular orbital (HOMO‐LUMO) gap of 0.55 eV for individual U_2_C@*I*_h_(7)‐C_80_, the band gap in 1D chains assembled by transition metal linker atoms are significantly widened. Specifically, 1D U_2_C@C_80_‐Cr, U_2_C@C_80_‐Mn, and U_2_C@C_80_‐Mo are direct semiconductors with band gap of 0.90, 1.43, and 1.07 eV, respectively, while 1D U_2_C@C_80_‐Ru has an indirect gap of 1.15 eV. The linkage of transition metal atoms has relatively large contribution to the electronic states near valence band maximum (VBM). Moreover, the electronic states of spin‐up (U_2_C@C_80_‐Cr), spin‐down (U_2_C@C_80_‐Mo) and spin‐cross (U_2_C@C_80_‐Mn/U_2_C@C_80_‐Ru) channels at the vicinity of Fermi level stem mainly from transition metal M and U atoms.

**Figure 2:**
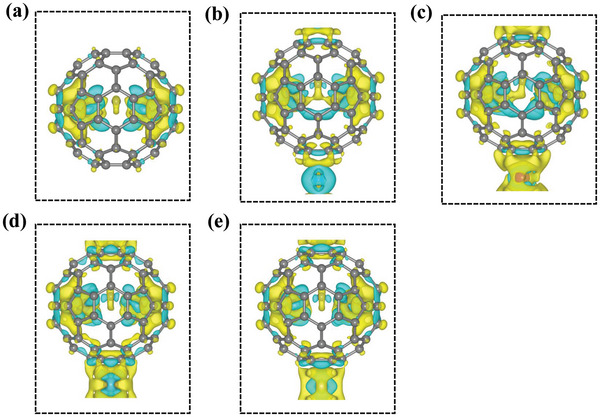
Differential charge density of a) U_2_C@*I*_h_(7)‐C_80_, b) U_2_C@C_80_‐Cr, c) U_2_C@C_80_‐Mn, d) U_2_C@C_80_‐Mo, and e) U_2_C@C_80_‐Ru 1D chains with an isosurface value of 0.03 e Å^−3^. Green and yellow denote charge depletion and accumulation, respectively.

The central symmetry of U_2_C units is further broken by the cooperative linkage of transition metal atoms in those 1D chains, leading to significantly spontaneous polarization (P_S_). The spontaneous polarization is computed using the modern polarization theory based on Berry‐phase method.^[^ ^29^ ^]^ As listed in **Table** **2**, the polarization of U_2_C@C_80_‐M chains are 27.77 pC m^−1^ for Cr, 34.60 pC m^−1^ for Mn, 27.34 pC m^−1^ for Mo, and 20.34 pC m^−1^ for Ru, respectively, which are about two to four times of 8.90 pC m^−1^ for the freestanding U_2_C@*I*_h_(7)‐C_80_. Such enhancement can be related to the increased bending degree of U_2_C units in 1D chains, i.e., U–C–U angle being 133.3°, 129.0°, 141.5°, and 145.1° in 1D U_2_C@C_80_‐M (M = Cr, Mn, Mo, and Ru), respectively, in comparison with that of metallofullerene molecule U_2_C@*I*_h_(7)‐C_80_ (145.6°). These effects can be understood by the interaction between fullerene U_2_C@*I*_h_(7)‐C_80_ and transition metal M atom. As illustrated by **Figure** **2**, the electrons transferred from M atom to C_80_ cages mean strong hybridization between them. Meanwhile, comparison with U_2_C@*I*_h_(7)‐C_80_, Bader charge and electron localized function (ELF) analysis further reveal that more negative charge is accumulated to the carbon atom in U_2_C trimer, resulting in sharper U–C–U angle and stronger electrostatic attraction between C and U atoms and thus enhancing the overall degree of polarization (Table S1 and Figure S3, Supporting Information).

**Table 2:** Computed spontaneous polarizations *P*s, switching barriers *E*_B_ at zero temperature, and fitted parameters in Equation (2). *A*, *B*, and *C* are used to describe the double‐well potential

	*P*_S_ [pC m^−1^]	*E*_B_ [meV atom^−1^]	*A* [10^−3^]	*B* [10^−6^]	*C* [10^−9^]
U_2_C@C_80_‐Cr	27.77	7.98	−6.97	20.01	−9.58
U_2_C@C_80_‐Mn	34.60	17.14	5.02	4.22	0.05
U_2_C@C_80_‐Mo	27.34	3.45	2.43	5.61	−1.10
U_2_C@C_80_‐Ru	20.34	2.74	2.10	3.83	4.74

**Figure 3:**
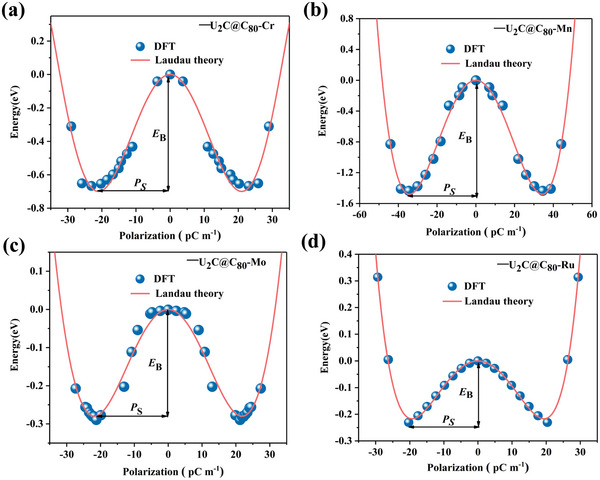
The double‐well potential as a function of the polarization for 1D a) U_2_C@C_80_‐Cr, b) U_2_C@C_80_‐Mn, c) U_2_C@C_80_‐Mo, and d) U_2_C@C_80_‐Ru chains. *E*_B_ and *P*s are the potential barrier and spontaneous polarization, respectively.

Here, U—C—U bond angle *θ* is defined to characterize the two distorted degenerate polar structures in Figure 1b, in which positive (*θ* > 0) and negative (*θ* < 0) angles correspond to ferroelectric phases (FE and FE′) with total polarizations (+*P* and −*P*), respectively, while the intermediate state with high symmetry nonpolar phase (*θ* = 0) is denoted as paraelectric phase (PE). The energy versus polarization *P* (or angle *θ*) is plotted in **Figure** **3** (Figure S4, Supporting Information), where the anharmonic double‐well energy curve is found to be consistent with the standard behavior for ferroelectrics.

According to the phenomenological Landau–Devonshire theory, the energy of a ferroelectric system can be described as a function of polarization *P* as

(2) $$E=\frac{A}{2}\;P^{2}+\frac{B}{4}P^{4}+\frac{C}{6}P^{6}$$

where *P* is the polarization value of the unit cell, and *A*, *B*, and *C* are constant coefficients. Equation (2) can be regarded as a Taylor series of local structural distortion up to sixth order. The fitted parameters *A*, *B*, and *C* are given in Table 2 and the double‐well potential as a function of polarization for U_2_C@C_80_‐M 1D chains are shown in Figure 3. One can be seen that two low‐energy FE and FE′ phases with opposite polarized directions are separated by a high‐energy PE phase. Using the nudged‐elastic band (NEB) method, the switching energy barriers between FE (FE′) phase and PE phase for all U_2_C@C_80_‐M chains were evaluated as 7.98 (M = Cr), 17.14 (M = Mn), 3.45 (M = Mo), and 2.74 (M = Ru) meV atom^−1^, respectively, which are lower than the theoretical values of reported 1D FE materials, such as nanowires of SbN (61 meV atom^−1^),^[^ ^30^ ^]^ BiN (28 meV atom^−1^),^[^ ^30^ ^]^ GeS (15 meV atom^−1^),^[^ ^31^ ^]^ SnS (5 meV atom^−1^).^[^ ^31^ ^]^ Evidently, such low barrier is beneficial to achieve high energy efficiency for ferroelectric devices, since lower external electric field is required to switch the direction of the electric polarization in the current systems. Additionally, other middle 3*d*/4*d* transition metal atoms, like Sc and V, have been considered as the linkers to construct U_2_C@C_80_‐Sc and U_2_C@C_80_‐V 1D chains, which also exhibit significant ferroelectricity (see Table S2, Supporting Information for details). Comparing to result with the 1D self‐assembled U_2_C@*I*_h_(7)‐C_80_ chain, namely U_2_C@‐C_80_−U_2_C@‐C_80_, the ferroelectricity of U_2_C@C_80_‐M chains bridged by the transition metal atoms is much stronger (Table S2, Supporting Information).

**Figure 4:**
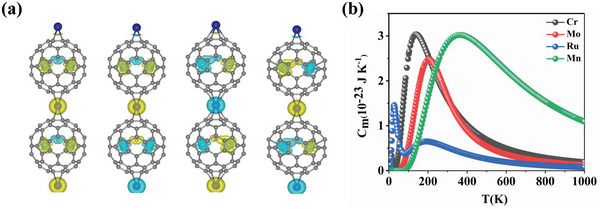
a) Spin densities of FM and three AFM configurations of U_2_C@C_80_‐M (M = Cr, Mn, Mo, and Ru) 1D chains with isosurface value of 0.05 e Å^−3^. Yellow and blue represent spin‐up and spin‐down densities, respectively. b) Heat capacity as a function of temperature for 1D chains.

After examining the ferroelectricity, the ferromagnetic behavior of these 1D chains has also been investigated. The spin density distributions in **Figure** **4**a indicate that the magnetism in 1D U_2_C@C_80_‐M is mainly contributed by transition metal M and U atoms. In detail, the on‐site magnetic moment of transition metal atom is 4.49 *µ*_B_ for Cr, 4.56 *µ*_B_ for Mn, 0.71 *µ*_B_ for Mo, and 0.90 *µ*_B_ for Ru, respectively, and each U atom possesses magnetic moment of about 1.66−2.12 *µ*_B_. There are only minor induced spin moments on the carbon atoms in U_2_C trimer and C_80_ cage, which could be ignored in the following discussion. In order to determine the magnetic ground states of 1D chains, we constructed various supercells with FM, and AFM states, as illustrated in Figure 4a. The exchange energy Δ*E*_ex_ is defined as

(3) $$\Delta E_{\mathrm{ex}}=E_{\mathrm{FM}}-E_{\mathrm{AFM}}$$

where *E*_FM_ and *E*_AFM_ are energies of the most stable FM and AFM configurations, respectively. According to the exchange energies calculated in Table S3 (Supporting Information), the exchange energies of 1D U_2_C@C_80_‐Cr and U_2_C@C_80_‐Ru are only −3.85 and 26.8 meV, respectively, meaning that the FM and AFM states are nearly degenerate. In contrast, the energy differences between FM and AFM states for U_2_C@C_80_‐Mn and U_2_C@C_80_‐Mo systems are 785.4 and −164.84 meV, respectively, indicating that the former has stable AFM ground state and the latter has robust FM ground state. Meanwhile, U_2_C@C_80_‐Sc, U_2_C@C_80_‐V, and U_2_C@‐C_80_−U_2_C@‐C_80_ 1D chains also show AFM ground states with energies difference between the FM and AFM states of 312, 230, and 170 meV, respectively (Table S2, Supporting Information).

The presence of magnetic anisotropy energy (MAE) is the key for the possibility of achieving stable long‐range magnetic order in 1D system at finite temperature.^[^ ^32^ ^]^ Therefore, we calculate the MAE of U_2_C@C_80_‐M 1D chains by considering the spin‐orbit coupling effect, as follows:

(4) $$\mathrm{MAE}=E_{∥}-E_{⊥}$$

where *E*_∥_ and *E*_⊥_ represent the energies of the system when the spin moment is parallel or perpendicular to the 1D chain plane, respectively. A positive MAE corresponds the out‐of‐plane preferential spin orientation without additional uncertainty for spin orientation, while the negative MAE represents in‐plane spin alignment. As seen from Table S3 (Supporting Information), U_2_C@C_80_‐M (M = Cr, Mn, and Mo) chains possess considerable out‐of‐plane MAE of 30 meV (Cr), 38 meV (Mn), and 21 meV (Mo), respectively, while U_2_C@C_80_‐Ru favors in‐plane anisotropy with MAE of −2 meV, implying that U_2_C@C_80_‐M chains can block the thermal fluctuation and stabilize their magnetic ground states.

We further estimate the magnetic transition temperatures for U_2_C@C_80_‐M (M = Cr, Mn, Mo, and Ru) 1D chains from the exact solution based on Ising model. The details are described in the Supplementary Materials. Here, we consider the exchange parameters *J*_1_ and *J*_2_ between the nearest‐neighboring U−U and the next‐nearest‐neighboring U–M, in which positive (negative) parameters correspond to FM and AFM state, respectively. As shown in Table S3 (Supporting Information), the exchange coupling parameters *J*_1_ and *J*_2_ for U_2_C@C_80_‐M 1D chains range from −37.63 to 20.73 meV and from −2.32 to 1.13 meV, respectively. The magnetic transition temperatures for U_2_C@C_80_‐M (M = Cr, Mn, Mo, and Ru) 1D chains are shown in Figure 4b. The ferromagnetic Curie temperature (*T*_C_) of U_2_C@C_80_‐Cr and U_2_C@C_80_‐Mo are 140 and 205 K, respectively. Compared with the previously reported SbN nanowire with both ferroelectric and ferromagnetic behavior (152 K),^[^ ^30^ ^]^ the Curie temperature of U_2_C@C_80_‐Mo chain is higher by 53 K. Meanwhile, the Néel temperature (*T*_N_) of 364 K for U_2_C@C_80_‐Mn chain is above room temperature. Additionally, for U_2_C@C_80_‐Ru system, the magnetic moment does not linearly decrease to zero with increasing temperature, and the magnetic transition behavior show ferrimagnetic (FiM) characteristics at 28 K. Hence, an appropriate choice of transition metal linker outside actinide endofullerene can steer a high Curie or Néel temperature.

**Figure 5:**
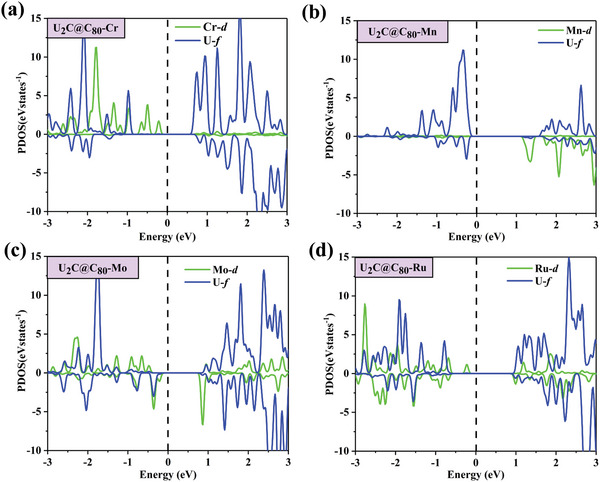
Orbital‐resolved density of states for a) U_2_C@C_80_‐Cr, b) U_2_C@C_80_‐Mn, c) U_2_C@C_80_‐Mo, and d) U_2_C@C_80_‐Ru 1D chains. The Fermi level as dashed line is shifted to zero.

To unveil the physical mechanism , we first analyze the atom projected density of states (PDOS) of these 1D chains in **Figure** **5**. The C_80_ skeleton exhibits delocalized *π*‐type states around Fermi levels, thereby providing a channel for long‐distance spin transport of 1D chains. For the VBM of U_2_C@C_80_‐Mo and U_2_C@C_80_‐Mn chains, U‐*f* orbitals couple with M‐*d* orbitals, and the conduction band minimums (CBM) are primarily attributed to M‐*d* orbitals. The VBMs and CBMs in U_2_C@C_80_‐Cr and U_2_C@C_80_‐Ru 1D chains originate from M‐*d* orbitals and U‐*f* orbitals, respectively. It should be noted that the long U–U distance in U_2_C units and spatially localized 5*f* orbitals lead to typically weak U–U direct exchange and antiferromagnetic coupling in U_2_C@*I*_h_(7)‐C_80_.^[^ ^12^ ^]^ Hence, the delocalized *d* orbitals of transition metal atoms could effectively extend highly localized 5*f* orbitals of U atoms, further achieving the cooperative competition mechanism between long‐range FM super‐exchange of U‐*f* orbitals and M‐*d* orbitals (U−M−U super‐exchange) and short‐range AFM direct exchange of U‐*f* orbitals and U‐*f* orbitals (U−U exchange).

For U_2_C@C_80_‐Mo 1D chain with robust ferromagnetism, significant hybridization between Mo‐*d* orbital and U‐*f* orbital near the Fermi level gives rise to large FM super‐exchange interactions, further weakening the direct U−U exchange. Similarly, the U−Cr−U super‐exchange interaction mediated by the Cr atom causes weak FM coupling in U_2_C@C_80_‐Cr chain, which is reflected by the partial overlap of Cr‐*d* orbital and U‐*f* orbital. On the contrary, for 1D U_2_C@C_80_‐Mn, the relatively smaller angle *θ* = 128.98° and shorter U…U distance 3.72 Å make the direct U−U exchange significantly stronger than the U−Mn−U super‐exchange, resulting in strong AFM character; where the overlap between the localized U‐*f* orbitals is enhanced, and the coupling between U‐*f* orbital and Mn‐*d* orbital is suppressed near the Fermi level. Compared with U_2_C@C_80_‐Mn chain, there is weaker Ru‐*d*−U‐*f* orbitals coupling near the Fermi level in U_2_C@C_80_‐Ru. Therefore, we speculate that the FiM coupling characteristic is consequence of the competition effect between Ru‐*d* with U‐*f* orbitals coupling and U‐*f* with U‐*f* orbital coupling. In a word, the inclusion of delocalized *d* orbitals of transition metal atoms would have different impacts on the 1D chain and attain extraordinary magnetic properties.

## 3 Conclusion

We have elaborately designed a series of fullerene‐based 1D chains with excellent ferroelectric and ferromagnetic properties. According to our first‐principles calculations, these U_2_C@C_80_‐M (M = Cr, Mn, Mo, and Ru) 1D chains have satisfactory thermal stability and excellent ferroelectricity. The cooperative linkage of transition metal atoms causes further breaking of the central symmetry of the endohedral U_2_C units, thereby leading to significantly larger spontaneous polarization than that of individual metallofullerene U_2_C@*I*_h_(7)‐C_80_. The low switching barriers between two FE states with opposite polarization endow these 1D chains potential for non‐volatile memory devices. Meanwhile, the magnetism in these 1D chains comes from U‐*f* orbitals and M‐*d* orbitals, and FM/FiM/AFM ground states are caused by competition between long‐range U−M−U interaction (M = Cr, Mn, Mo, and Ru) and short‐range U−U exchange interaction. The tunable magnetic transition temperature of 1D chains assembled by transition metal linker atoms opens the doorway to novel low‐dimensional spintronic devices. Our results show the feasibility and prospect of endohedral fullerenes in nanoscale multiferroic devices. Since low‐dimensional multiferroic materials are still extremely scarce, the present findings are anticipated to stimulate more theoretical and experimental efforts on 1D multiferroic materials.

## 4 Experimental Section

First‐principles calculations were performed with spin‐polarized density functional theory implemented in the Vienna Ab initio Simulation Package .^[^ ^33^, ^34^ ^]^ The Perdew−Burke−Ernzerhof ^[^ ^35^ ^]^ parameterization within the generalized gradient approximation (GGA) was used to describe the exchange–correlation interactions. The plus *U* method (GGA+*U*) was applied to appropriately describe the on‐site Coulomb interactions for 5*f* orbitals of U atom and 3*d/*4*d* orbitals of Cr, Mn, Mo, and Ru atoms, respectively.^[^ ^36^, ^37^, ^38^, ^39^, ^40^, ^41^ ^]^ The energy cutoff of plane‐wave basis was 500 eV. A vacuum space with 30 Å thickness was added to eliminate the interaction between two adjacent layers and the Brillouin zone was sampled with 1 × 1 × 3 k‐points. Both atomic positions and lattice constants were fully relaxed with the converge criteria of 10^−6^ eV for energy and 10^−2^ eV Å^−1^ for force, respectively. Based on equilibrium geometries, the HSE06 was also utilized to obtain more accurate description of the electronic properties. The magnitude of polarization was calculated by the standard Berry phase in modern polarization theory.^[^ ^42^ ^]^ To assess the stability of these 1D chains, AIMD simulation within NVT canonical ensemble was performed for up to 7.5 ps with 1.5 fs time step.

## Conflict of Interest

The authors declare no conflict of interest.

## Supporting information

Supporting Information

## Data Availability

The data that support the findings of this study are available from the corresponding author upon reasonable request.
