# Supplementary material for: Coexistence of Ferroelectricity and Ferromagnetism in Fullerene‐Based One‐Dimensional Chains
Source: Adv Sci (Weinh). 2023 May 10;10(21):2301265. doi: 10.1002/advs.202301265 (PMC10375193; doi:10.1002/advs.202301265)
Supplement: Supplementary file 1 — Supporting Information [file ADVS-10-2301265-s001.pdf]

ADVANCED SCIENCE
Open Access

Supporting Information

for *Adv. Sci.*, DOI 10.1002/advs.202301265

Coexistence of Ferroelectricity and Ferromagnetism in Fullerene-Based One-Dimensional Chains

Yang Zhao, Yu Guo, Yan Qi, Xue Jiang, Yan Su and Jijun Zhao**

Supporting Information

Coexistence of Ferroelectricity and Ferromagnetism in Fullerene-based one-dimensional chain

Yang Zhao, Yu Guo, Yan Qi, Xue Jiang, Yan Su, Jijun Zhao**

Calculation methods of Curie temperatures and Néel temperature

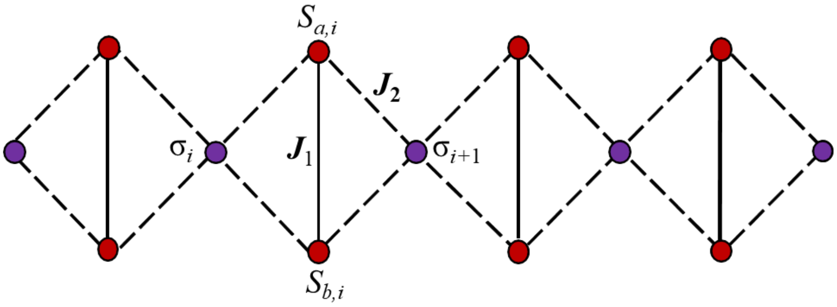

We consider a symmetric Ising diamond chain model to describe $U_2C@C_{80}$-M (M = Cr, Mn, Mo, and Ru) 1D chains magnetic properties. Each plaquette Hamiltonian unit H_i contains all the interaction terms associated with these two kinds of Ising spins:

$$H_i = -J_1 S_{\mathrm{a,i}} S_{\mathrm{b,i}} - J_2(\sigma_i + \sigma_{i+1})\left(S_{a,i} + S_{b,i}\right) \tag{S1}$$

where J_1 and J_2 are first, second nearest-neighbor exchange parameters, respectively, $S_{\mathrm{a,i}}$, $S_{\mathrm{b,i}}$are the magnetic moments of U atoms at site i; σ_i and $\sigma_{i+1}$are the magnetic moments of transition metal atoms M at site i and site i+1.

For a class of this simplified Ising spin system, the decoration-iteration transformation[1] and the transfer-matrix technique are both commonly used methods to obtain their corresponding exact results. And the obtained exact solutions can shed light on the properties of real magnetic materials, even yielding quantitative agreement with the experimental representatives in some aspects. In this paper, the transfer-matrix method is employed to rigorously determine the magnetic specific heat. In view of the solution procedure described in detail in many Refs.,[2-4] we will

not repeat the technical details here again.

We define the Bader charge difference (ΔQ) between a specific atom on the $U_2C@C_{80}$-M 1D chains and the corresponding atom on the metallofullerene molecule $U_2C@I_h(7)$-C_{80} as follow:

$$\Delta Q = Q_{U_2C@C_{80}\text{-M}} - Q_{U_2C@I_h\text{-}C_{80}} \quad \text{(S2)}$$

where $Q_{U_2C@C_{80}\text{-M}}$ and $Q_{U_2C@I_h\text{-}C_{80}}$ are the Bader charge of a specific atom on the $U_2C@C_{80}$-M 1D chains and metallofullerene molecule $U_2C@I_h(7)$-C_{80}, respectively.

Table S1. The Bader charge differences (ΔQ) from C and U atoms of 1D chain U_2C trimer.

	C	U
$U_2C@C_{80}$-Cr	–0.06	–0.08
$U_2C@C_{80}$-Mn	–0.2	–0.13
$U_2C@C_{80}$-Mo	–0.19	–0.12
$U_2C@C_{80}$-Ru	–0.08	–0.08

Table S2. Computed lattice parameters (a), U–C–U angles (θ), band gap (E_g^{HSE}), spontaneous polarizations (Ps), switching barriers (E_B), magnetic ground states (GS), exchange energies (ΔE_{ex}) of $U_2C@C_{80}$-Sc, $U_2C@C_{80}$-V, and $U_2C@$-C_{80}–$U_2C@$-C_{80} 1D chains.

	A [Å]	θ [°]	E_g^{HSE} [eV]	P_S [pC m^{-1}]	E_B [meV atom^{-1}]	GS	ΔE_{ex} [meV]
$U_2C@$-C_{80}–$U_2C@$-C_{80}	10.31	145.4	0.47	12.68	2.74	AFM	170
$U_2C@C_{80}$-Sc	11.92	131.4	0.54	29.91	11.31	AFM	312
$U_2C@C_{80}$-V	11.69	133.7	1.19	33.24	12.50	AFM	230

Table S3. Magnetic ground states (GS), exchange energies (ΔE_{ex}), exchange coupling

parameters (J_1 and J_2), and magnetic anisotropy energies (MAE) for $U_2C@C_{80}$-M (M = Mn, Mo, and Ru) 1D chains.

	GS	ΔE_{ex} [meV]	J_1 [meV]	J_2 [meV]	MAE [meV]
$U_2C@C_{80}$-Cr	FM	–3.85	14.80	0.07	30
$U_2C@C_{80}$-Mn	AFM	785.40	–37.63	–0.99	38
$U_2C@C_{80}$-Mo	FM	–164.84	–8.92	31.13	21
$U_2C@C_{80}$-Ru	FiM	26.80	20.73	–2.32	–2

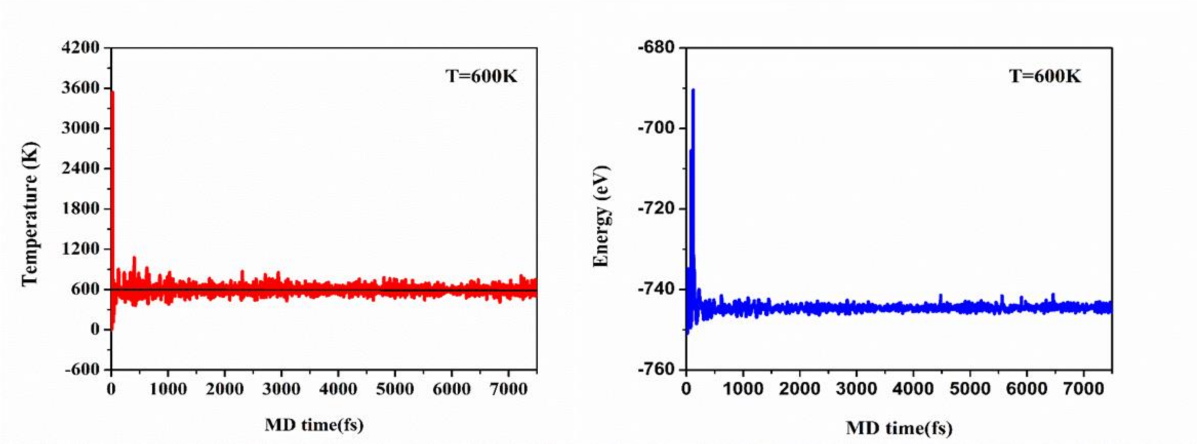

Figure S1. Variations of temperature and energy with the time of AIMD simulation for $U_2C@C_{80}$-Mo 1D chain at 600K.

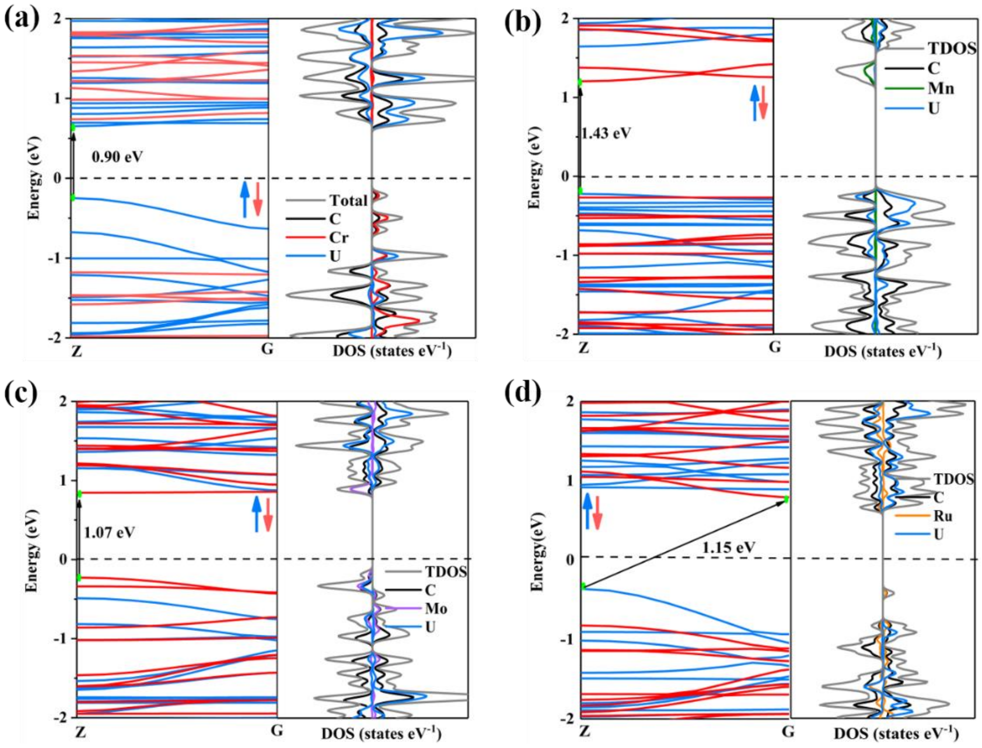

Figure S2. Band structures and density of states (DOS) of $U_2C@C_{80}$-M (M = Cr, Mn, Mo, Ru) 1D chains. The dashed line shows the Fermi level that is shifted to zero.

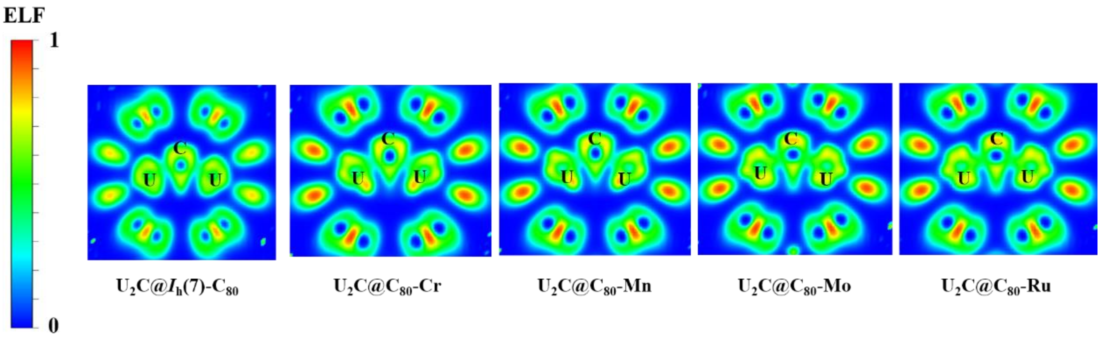

Figure S3. Contour plots of ELF for $U_2C@I_h(7)$-C_{80} and $U_2C@C_{80}$-M (M = Cr, Mn, Mo, and Ru) 1D chains on the (100) plane.

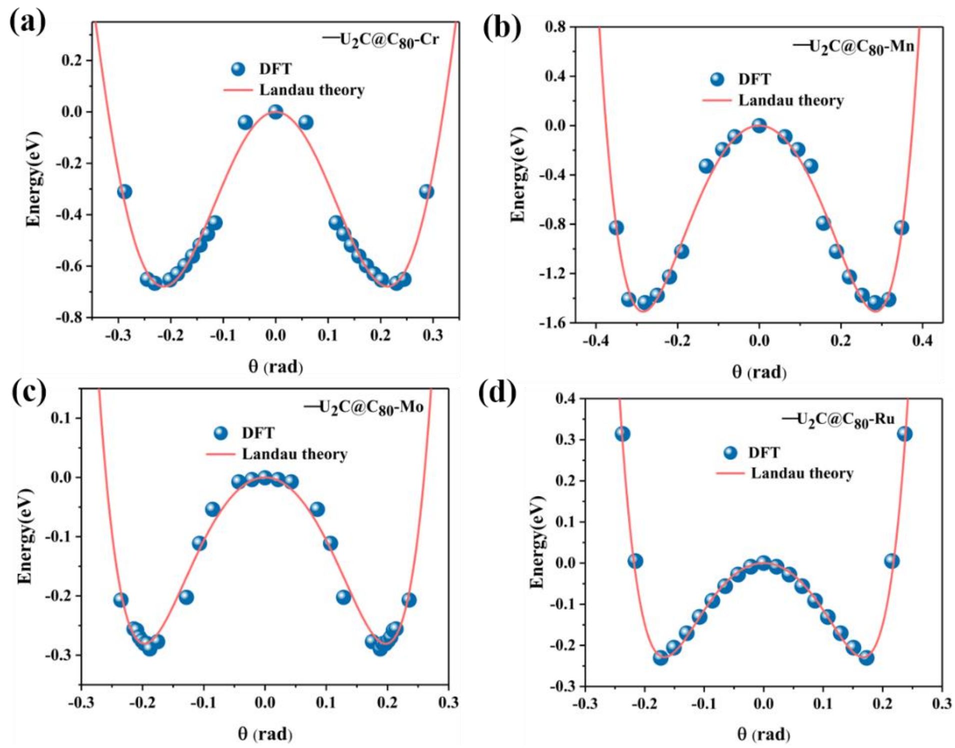

Figure S4. A double-well potential as a function of the angle for (a) $U_2C@C_{80}$-Cr, (b) $U_2C@C_{80}$-Mn, (c) $U_2C@C_{80}$-Mo, and (d) $U_2C@C_{80}$-Ru 1D chains, respectively.

References

(1) Gálisová. *Matter. Phys.* **2014,** *17*, 13001.

(2) O. Rojas, M. Rojas, N. S. Ananikian, S. M. de Souza, *Phys. Rev. A* **2012,** *86*, 042330.

(3) Y. Qi, A. Du, *Phys. Phys. Status Solidi B* **2014,** *251*, 1096.

(4) D. Antonosyan, S. Bellucci, V. Ohanyan, *Phys. Rev. B* **2009,** *79*, 014432.
